# ‘Diabetes is a gift from god’ a qualitative study coping with diabetes distress by Indonesian outpatients

**DOI:** 10.1007/s11136-019-02299-2

**Published:** 2019-09-23

**Authors:** Bustanul Arifin, Ari Probandari, Abdul Khairul Rizki Purba, Dyah Aryani Perwitasari, Catharina C. M. Schuiling-Veninga, Jarir Atthobari, Paul F. M. Krabbe, Maarten J. Postma

**Affiliations:** 1 Department of Pharmacy, Unit of Pharmacotherapy, Epidemiology & Economics (PTE2), Faculty of Science and Engineering (FSE), University of Groningen, Groningen, The Netherlands; 2 Diseases Preventing and Control Division, Banggai Laut Regency Health, Population Control & Family Planning Service, Banggai Laut Local Government, Central Sulawesi Indonesia; 3 Present Address: Department of Health Sciences, University of Groningen, University Medical Center Groningen, Groningen, The Netherlands; 4 Institute of Science in Healthy Ageing & HealthcaRE (SHARE), University of Groningen, University Medical Center Groningen (UMCG), Groningen, The Netherlands; 5 Department of Public Health, Faculty of Medicine, Universitas Sebelas Maret, Jl. Ir. Sutami 36A, Surakarta, 57126 Indonesia; 6 Department of Pharmacology and Therapy, Faculty of Medicine, Universitas Airlangga, Surabaya, Indonesia; 7 Faculty of Pharmacy, University of Ahmad Dahlan, Yogyakarta, Indonesia; 8 Department of Pharmacology and Therapy, Faculty of Medicine, Public Health and Nursing, Universitas Gadjah Mada, Yogyakarta, Indonesia; 9 Department of Epidemiology, University Medical Center Groningen, University of Groningen, PO Box 30.001, 9700 RB Groningen, The Netherlands; 10 Clinical Epidemiology and Biostatistics Unit, Faculty of Medicine, Public Health and Nursing, Universitas Gadjah Mada, Yogyakarta, Indonesia; 11 Department of Economics, Econometrics & Finance, Faculty of Economics & Business, University of Groningen, Groningen, The Netherlands; 12 Department of Medical Microbiology, University Medical Center Groningen (UMCG), University of Groningen, Groningen, The Netherlands

**Keywords:** Diabetes distress, Indonesian T2DM, Spirituality, Housewives

## Abstract

**Background:**

More than two-thirds of patients diagnosed with type 2 diabetes mellitus (T2DM) in Indonesia encounter medical-related problems connected to routine self-management of medication and the social stigma related to T2DM. The current study aims to explore distress and coping strategies in Indonesian T2DM outpatients in a Primary Healthcare Centre (PHC) in Surabaya, East Java, Indonesia.

**Methods:**

We conducted a qualitative study using two different data collection methods: focus group discussions and in-depth interviews. The guideline of interviews and discussions were developed based on seventeen questions derived from the DDS17 Bahasa Indonesia (a Bahasa Indonesia version of the Diabetes Distress Scale questionnaire), which covered physician distress domain, emotional burden domain, regimen distress domain and interpersonal distress domain.

**Results:**

The majority of the 43 participants were females and aged 50 or older. Our study discovered two main themes: internal and external diabetes distress and coping strategies. Internal diabetes distress consists of disease burden, fatigue due to T2DM, fatigue not due to T2DM, emotional burden (fear, anxiety, etc.) and lack of knowledge. Internal coping strategies comprised spirituality, positive attitude, acceptance and getting more information about T2DM. External diabetes distress was evoked by distress concerning healthcare services, diet, routine medication, monthly blood sugar checks, interpersonal distress (family) and financial concern. External coping strategies included healthcare support, traditional medicine, vigilance, self-management, social and family support and obtaining information about health insurance.

**Conclusion:**

Our study shows that for Indonesian T2DM-patients, spirituality and acceptance are the most common coping mechanisms for reducing DD. Furthermore, our study revealed an overall positive attitude towards dealing with T2DM as well as a need for more information about T2DM and potential coping strategies. Finally, an important finding of ours relates to differences in DD between males and females, potential DD associated with health services provision and the specific challenges faced by housewives with T2DM.

## Introduction

Indonesia is one of the Southeast Asian countries with the highest rate of new DM cases [1, 2]. The Ministry of Health of The Republic of Indonesia reported that the prevalence of DM patients had escalated from 1.1% in 2007 to 2.1% in 2013 [3]. It was also reported that there were 10 million people living with DM in Indonesia in 2015. Indonesian ranked sixth out of the 10 countries with high rates of DM cases, worldwide [1]. Among all types of DM, 90% of patients were type 2 diabetes mellitus (T2DM) [4].

T2DM not only affects the physical functions of the patients but it can also increase the risk of developing mental health problems, such as depression and diabetes distress (DD) [5]. The term ‘distress’ was introduced in the 1970s by Hans Selye, a Hungarian physiologist, as a continuous measure within his theory on ‘the non-specific response of the body to any demand for change’ [6–8]. In short, the term is used to describe a person who experiences a problem or is in an uncomfortable situation in daily life [9]. Furthermore, the term distress is mainly used in the context of chronic diseases such as T2DM.

Each country has its own socio-demographic characteristics, clinical conditions and other related factors in determining DD. In the United States of America and South Africa studies, they found similar DD-related factors, such as younger age and lower levels of public healthcare support [10, 11]. Two studies in Malaysia revealed that being female, having higher levels of systolic blood pressure [12], having a level of HbA1c more than 8.5%, the presence of comorbid, lifestyle and family history of psychiatric illness were all highly associated with a high level of DD [13]. Furthermore, an Iran study reported that being female and the level of education to be the significant predictors of DD [14]. Nonetheless, the complexity of DD in T2DM is not limited to the socio-demographic and clinical conditions mentioned above, but several other factors such as chronic stress [15], lack of knowledge [16], personal attitude [17], self-management (diet, exercise and blood sugar checks) [18] and financial concerns [16] affects DD too.

Several studies outline various strategies to cope with DD for T2DM outpatients, such as spirituality [19], self-management [18] and family and social support [20–24]. Spirituality is a coping mechanism that is used in several countries, such as Iran [25], Georgia [26] and Malaysia [27]. In Indonesia however, the connection between DD and coping mechanisms is poorly understood. This study explores distress and coping strategies in Indonesian T2DM outpatients in a Primary Healthcare Centre (PHC) in Surabaya, East Java, Indonesia.

## Methods

### Research context

East Java province consists of 29 regencies and nine cities, with 662 sub-districts. It covers a total area of approximately 47,800 km^2^, and the population was nearly 39 million in 2014. The study was conducted in a PHC setting in Surabaya, the capital city of East Java province. Moreover, Surabaya is the second largest city in Indonesia after Jakarta and one of the national health referral centres in Indonesia. East Java has 229 public hospitals, 90 private hospitals and 960 PHCs [28]. We chose to collect data in Surabaya mainly because East Java is one of the provinces with the highest number of diabetes patients [3] and the location where we carried out this study was considered as the most successful health facility in implementing the Indonesian Prolanis diabetes programme.

The Prolanis members are involved in healthcare support activities, including a diabetes club for weekly physical exercise. Prolanis is also involved in the dissemination of information about T2DM to general practitioners (GPs) or internists and information sharing among its members. From 2014 until June 2018, the number of Prolanis members has increased threefold from 114,361 to 345,995 [29]. In addition, surveys from January 2014 to September 2017 of 4800 Prolanis participants proved that the more active Prolanis participants who participated in Prolanis activities had lower number of visits to health facilities (secondary and tertiary) and lower costs of health services [29]. T2DM outpatients in Surabaya are assigned to a PHC selected by the Indonesian Health Insurance scheme provided by the BPJS/Badan Penyelenggara Jaminan Sosial (Social Security Administrative Agency). The BPJS runs many programmes to support diabetes care, including Prolanis.

### Study design

**Fig. 1:**
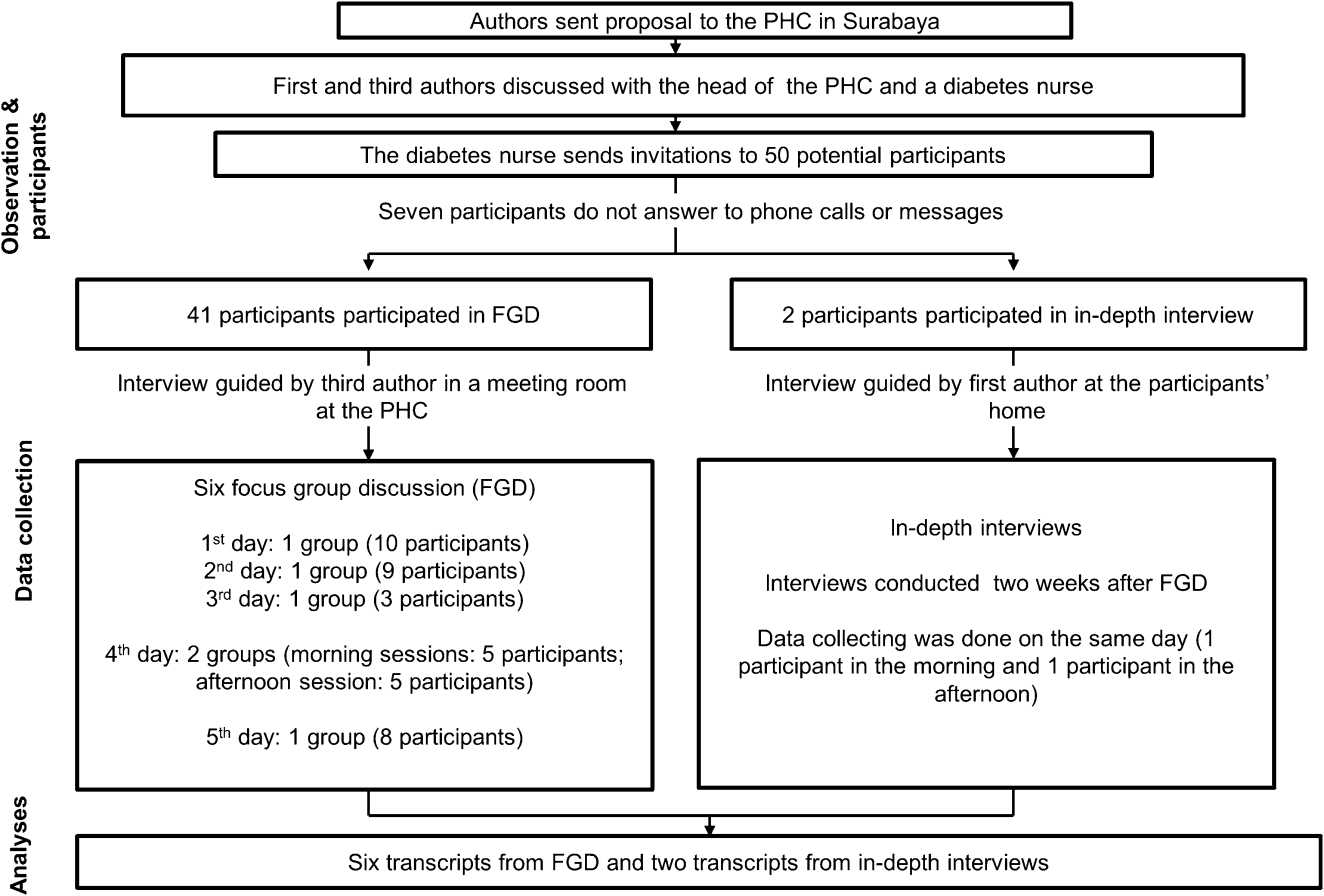
Flowchart of the study procedure

We used COREQ (COnsolidated criteria for REporting Qualitative research) to support the comprehensive reporting in our study [30]. The qualitative research applies a qualitative phenomenology approach whereby the participants describe their everyday life [30, 31]. In the interviews, the open questions were developed from DDS17 Bahasa Indonesia [32]. DDS17 Bahasa Indonesia consisted of 17 questions, which covered four domains. Firstly, the physician distress domain is covered (four questions), that provides a description of participant opinion toward knowledge and concern of the treating physician. Secondly, the emotional burden domain covers five questions on the concerns and fears of T2DM patients with T2DM complications. Thirdly, the regimen distress domain (four questions) aims to measure participant difficulty in the management of T2DM therapies. Finally, the interpersonal distress domain (three questions) concerns support of family and colleague of patients with T2DM. The details are presented in Appendix 1. Data collection was conducted in June 2015 which comprised of the following steps: submission of the research proposal to the intended PHC, participants selection, FGDs and in-depth interviews. A flowchart of our study procedure is presented in Fig. 1.

### Research team and reflexivity

The qualitative study was initiated by an FGD and carried out by the third author (male) who was also a medical doctor in Surabaya. The third author was trained as a research assistant by the first (male) and second author (female) before the initial phase of this study. The in-depth interviews were conducted by the first author who holds a master degree in hospital management and is also a pharmacist in a public hospital in Central Sulawesi. The first author had experience in qualitative research and had participated in several courses and workshops in qualitative research. Interviewers introduced the research objectives and the roles of the participants at the beginning of both FGDs and in-depth interviews, and the participants were given a chance to ask questions. The interviewers and the participants had not known each other before. Reflexivity applies to the whole team, not only the ones who conducted the data collection.

### Observation, participants and data collection

The feasibility of conducting this study had been discussed with the head of the PHC and a diabetes nurse and resulted in a list of 50 potential participants who could participate in the FGDs (purposive sampling). The invitation, which was sent by mail, contained the study objectives, the location of the FGD and five options on when to conduct the FGD. Furthermore, 41 participants confirmed their attendance for the FGDs by contacting the diabetes nurse in charge. Time to conduct the FGD was arranged based on participants’ time preferences. There were six FGDs sessions planned with five-time slots. Within the FGD, two study nurses assisted the interviewer for administrative purposes.

The first author sent messages to the nine other participants who were absent from the FGD for the in-depth interviews. Two potential female participants responded, and they said they wished to be interviewed at home. Data collection was done on the same day in which one of the participants was interviewed in the morning, and the other participant was interviewed after lunch. We used the same interview guidelines for the FGDs and in-depth interviews.

Overall, out of the 43 participants, none of them had a university degree. Participants lived in the vicinity of the PHC location. We recorded all the interviews using audio recordings. All the participants permitted the photo session during the interview process, and the participants in the FGD sessions also signed the attendance list. The FGDs lasted for about 20–50 min, and the in-depth interviews lasted around 10–15 min. The time was measured from the point when the participants agreed to be recorded.

### Data processing and analysis

Thematic analysis was performed for coding, analysing (categorising) and reporting pattern (themes) within the data [33]. The first and third authors read the transcripts and labelled the most meaningful statements independently from the coding process. Extracting not only the obvious meaning (the literal sense of the words) but also the latent meaning (the potentially hidden content) of statements in the transcripts and reducing the number of coding items by combining or deleting redundant codes, were two crucial steps in the coding process. The coding results in Bahasa Indonesia were discussed among the Indonesian authors. All the fixed codes were grouped into several categories based on their similarity. The categories (in English) were then sent to all the authors, who were asked to arrange them into themes (superordinate and subordinate). Discrepancies in the authors’ allocation of categories to themes were settled by consensus. We used Open Code 3.4 (open source software developed by the Department of Epidemiology and Global Health, Umeå University, Sweden). We also performed the analysis to see the relationship between the sex and age of participants with DD using IBM SPSS Statistics for Windows, version 23 (SPSS Inc., Cambridge, MA, USA).

### Ethics

Ethical approval was obtained from the ethics committee of the Faculty of Medicine, Universitas Gadjah Mada in Yogyakarta, Indonesia (document number KE/FK/1188/EC, 12th November 2014, amended 16th March 2015). Participants enrolled in the study were informed verbally about the study content. Participants willing to join the study signed an informed consent form in which they also gave their permission to be recorded (FGD or in-depth interview). All the participants were kept completely anonymous.

## Results

### Participant characteristics

In this study, all the female participants informed us that they were housewives. Of the male participants, only one participant was still an active employee the seven others participants were pensioners. No information was available about the duration of T2DM disease because most participants could not remember the date they were first diagnosed. Table 1 describes the socio-demographic characteristics of all the participants.

**Table 1:** Demographic details of participants (*n *= 43)

Description	Participants, *n* (%)
Age	
≤ 56	11 (26)
> 56	32 (74)
Sex	
Male	8 (18)
Female	35 (82)
Level of education	
None	2 (5)
Primary (6 years)	9 (21)
Secondary (9 years)	13 (30)
Senior secondary (12 years)	19 (44)
Types of treatment	
Diet	3 (7)
Oral antidiabetic drugs	31 (72)
Insulin (mono or combination)	4 (9)
Insulin + OAD	5 (12)
Number of complications and comorbidities	
Without complications	17 (40)
1 complication (no comorbidity)	15 (35)
2 or more complications	8 (18)
Comorbidity (breast cancer and gastritis)	3 (7)
Caregivers	
None (alone)	24 (56)
Husband/wife	6 (14)
Son/daughter	12 (28)
Mother/father	1 (2)
Occupations	
Active employee	1 (2)
Retired	7 (16)
Housewife	35 (82)

**Fig. 2:**
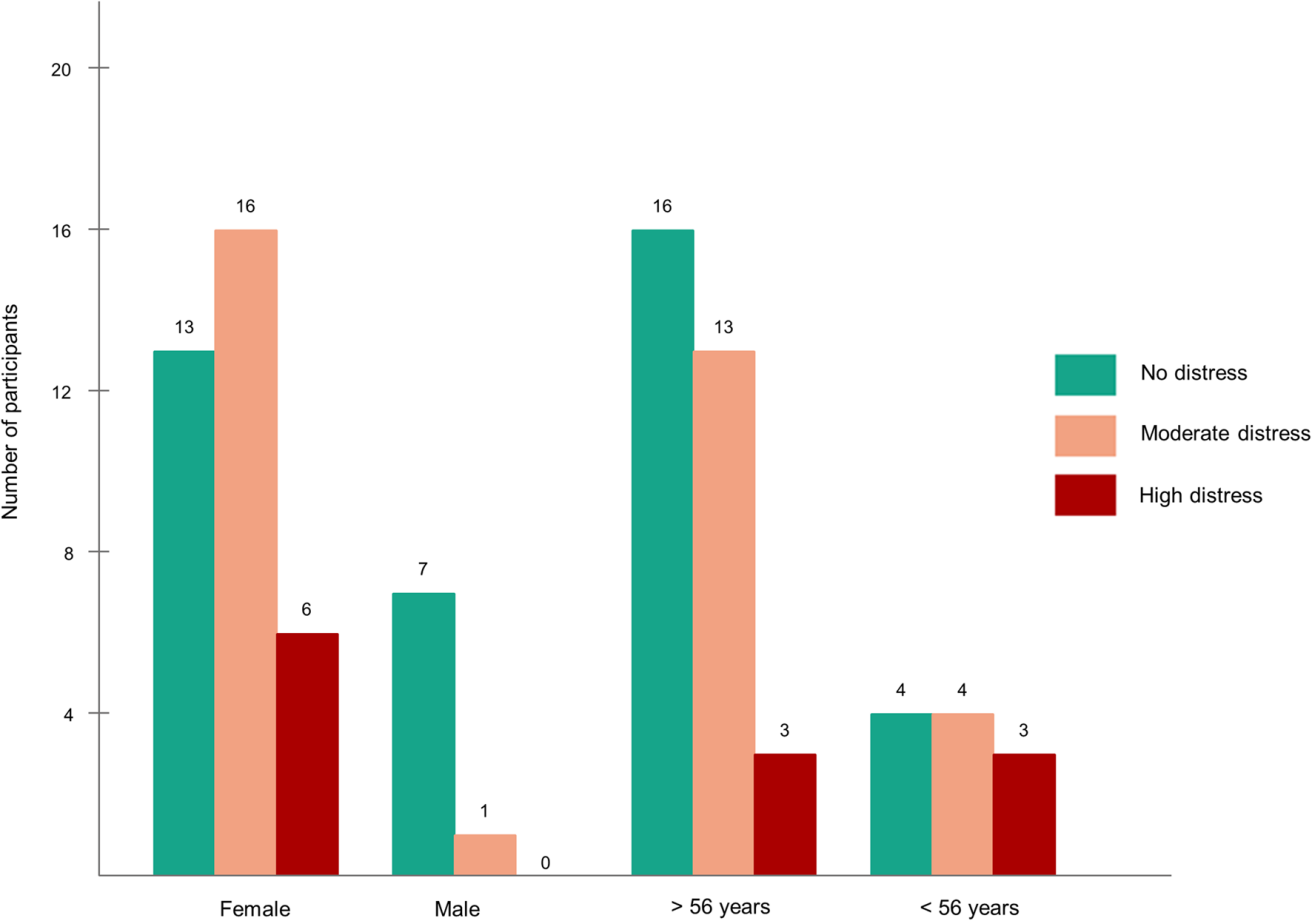
The number of participants with three categories of diabetes distress by sex and age

Based on socio-demographic characteristics, it was found that most of the participants involved in this study were female (82%). The following Fig. 2 described the relationship between sex and age with DD (none, moderate DD and high DD) [34]. The result of our analysis revealed that DD was experienced more by female participants compared to male participants (*p *=* .*034). Sixteen female participants were seen as experiencing moderate distress, and six female participants can be seen as experiencing high distress. On the other hand, there was only one male participant who appeared to experience moderate distress). Furthermore, In Indonesia, the pension age for civil servants is 56 years old, so we divided the participant’s age into two categories (more and less than 56 years). There was no significant relationship between age and DD (*p *=.326).

### Interview results

Eight transcripts were developed from six FGDs and two in-depth interviews. Some coding revision was necessarily carried out to achieve agreement amongst the Indonesian authors. The results from the coding process were 298 initial codes. After further discussion, we agreed on 98 relevant codes grouped into 21 overall categories. Table 2 depicts some examples of the process of coding from meaning units to categories. The categories were grouped into themes by consensus amongst the authors, as described in the methodology. We did not give the participants the opportunity to check the transcripts.

**Table 2:** Examples of how we label sentences based on their sense unit

Questions (original DDS domains)	Sense unit in English (participants)	Coding	Categories
Feeling that diabetes is taking up too much of my mental and physical energy every day (regimen distress)	No problem. That is one of the consequences of living with diabetes. 3 J (number of calories, schedule and type of food) foods must be consumed. Must be punctual with mealtimes	-Consequence of daily routine -Self-management -Effectiveness of 3 J programme -Promotion easy to remember and practice	Emotional burden Disease burden Healthcare support
Feeling angry, scared or depressed when I think about living with diabetes (emotional burden)	I must enjoy this condition. If I think about this more, I will be distressed. So just enjoy (M, f, 50, 10) ‘I still feel grateful to God for giving me this disease.’ (M, female, aged 56)	- Stress management -Spirituality -Acceptance	Acceptance Spirituality
Feeling that diabetes controls my life (emotional burden)	No problem, It is not a problem for people with diabetes to eat rice cooked yesterday. It is also not a problem to eat freshly-cooked rice. I ate that yesterday, and I am okay (S, f, 54, 10)	-Knowledge of food management -Belief in community rumours -Inadequate knowledge	Diet Lack of knowledge
Feeling that my doctor does not know enough about diabetes and diabetes care (physician distress)	The doctor does not explain how the medicine should be taken. Perhaps it is dangerous I am confused! (S, m, 55, 5)	-Impact of physicians’ explanations -Misunderstanding -Unclear information	Distress concerning healthcare service
Feeling that I do not have a doctor whom I can regularly see about my diabetes (physician distress)	Hmm, there are so many patients for the doctor to check I went to a private doctor, and it was good. I had to pay 100,000 rupiahs, but I could ask as many questions as I needed	-Difficulty_health facilities -Action_pay more -Action_choose private physician -Desire_detail diagnose -Desire_consultation time -Expectation_comfortable -Desire_curious about disease -Desire_dare to take a decision -Desire_second opinion from another physician	Distress concerning healthcare service Financial concerns
Feeling that I am not testing my blood sugar frequently enough (regimen distress)	I wish I could have that once a week, but it is forbidden (E, f, 45, 5)	-Consequences -Fear_uncontrolled blood sugar	Diet Disease burden
Feeling that I am often failing in my diabetes regimen (regimen distress)	‘I really want to eat *mas*. I am afraid of eating *kikil,* but I eat ice cream, he….he…’ (K, female, aged 56, 2)	-Desire_food management (which one better) -Diet	Diet Lack of knowledge
Feeling that friends or family do not give me the emotional support I would like (interpersonal distress)	My mother has ten siblings, and all of them suffer from diabetes, so I know a lot about the disease I am so upset at being ignored. Sometimes I need to get insulin while I am working; then, I go myself Sometimes it makes me angry. I go by myself; I can do it (M, f, 60, 5)	-Consequence of disease_genetic -Knowledge_insulin is the best -Believing rumours about insulin better than OAD -Knowledge_OAD due to kidney diseases	Interpersonal distress (family) Vigilance

**Fig. 3:**
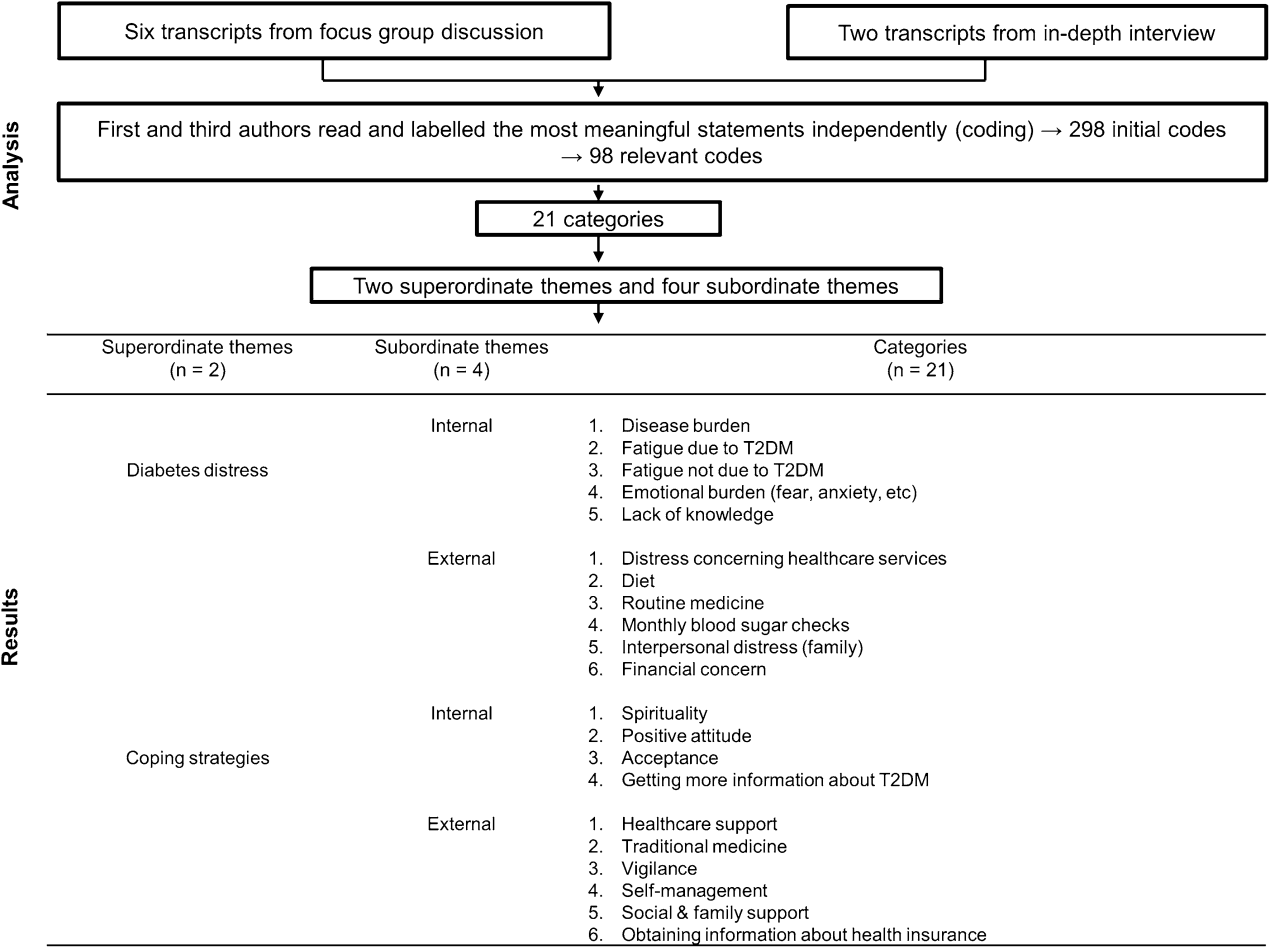
Analysis and results of the interviews

Themes were formulated to link the meaning of categories. The study revealed two superordinate themes, four subordinate themes and 21 categories (Fig. 3).

#### Internal diabetes distress

Our study showed that males and females have different perspectives. The female participants stated that they encountered difficulties in their daily activities. The participants who were housewives felt unsettled because they had to take care of themselves and their families including house cleaning and meal preparation. They indicated that some problems stemmed from having to prepare food for the family that would also be suitable for T2DM patients. Moreover, the female participants felt that the burden of T2DM and its complications, on top of their daily responsibilities, accounted for the primary cause of their psychological fatigue. In contrast, male participants, especially elderly men, were not as concerned about their disease. They preferred to spend their time at work or on hobbies such as fishing and visiting friends and neighbours. The internal DD were disease burden, fatigue due to T2DM, fatigue not due to T2DM, emotional burden (fear, anxiety, etc.) and a lack of knowledge.

##### Disease burden

Having a headache was the most common symptom reported by the female participants, and this symptom served as a natural alarm. When headaches occurred, this triggered self-evaluation, regardless of whether the headaches were caused by a failure to take medicine or the consumption of food unsuitable for T2DM.

If my blood sugar increases, my body hurts. Sometimes, the pain is in my legs. Sometimes I feel a headache. I asked my doctor once how this occurs. My doctor said that my levels of cholesterol and uric acid were normal and that my headaches were due to my high blood sugar levels. (I42; female, aged 37).

##### Fatigue due to T2DM

Almost all the female participants stated that they tried to hide their physical and psychological fatigue. Two housewives whose children did not live with them said that revealing their disease to their families could not reduce their disease burden and in fact added more stress in the household. They felt that expressing their T2DM burden would interrupt their children’s studies. In conclusion, several housewife participants kept the burden of T2DM to themselves.

I feel so tired, but I do not tell the family. (I26; female, aged 69).

Most of the female participants were forced to restrict their activities because of T2DM, especially activities away from home. Moreover, they reported that when they were at home, they also felt tired, although they did not perform any daily activities.

Since I have had diabetes, I always feel tired in my daily activities, and even though I just stay quiet, I still feel exhausted. (I11, female, aged 66).

##### Fatigue not due to T2DM

Most of the male participants stated that their source of daily fatigue depended on the type of activities they undertook. Most of the participants in this group also reported no perceived difference in the fatigue before and after suffering from T2DM.

Even before I was diabetic, I would have felt as tired because I have so much work to do. (I43; male, aged 48).

##### Emotional burden (fear, anxiety, etc.)

Participants diagnosed with the T2DM experienced psychological burden, especially those with no family history of T2DM. Most participants reported that they did not recall exactly when they first developed T2DM. Some stated that they went to a particular healthcare facility due to other diseases, such as fever or soreness, even though those symptoms only lasted for a few days.

When I was diagnosed with diabetes, I did not eat for three weeks. My blood sugar reached 430mg/dL. I had problems eating, and I could not sleep either. (I42; female, aged 37).

The psychological burden of T2DM can also have impacts on physical conditions which was a symptom that affected female participants in particular. They felt torn between their responsibilities and obligations as a mother or a wife, as well as being affected by the general negative stigma that surrounds T2DM.

I was diagnosed with diabetes the first time I had my blood sugar checked at the hospital. I was very stressed. I have lost weight since then. (I3; female, aged 56).

Another impact of having T2DM was fear which appeared in several examples. Such fears included the fear of not getting to see their children grow up and the fear of not being able to fulfil their children’s needs.

My children are still so young […] (I42; female, aged 37).

Furthermore, in these cases, the wife depended on the husband to remind her to take her medication, and therefore the husband was seen as a reliable support.

My husband reminds me never to forget to take medicine. Sometimes my husband helps me to inject the insulin. (I42; female, aged 37).

The emotional burden for males with T2DM is slightly different from females. Male participants felt that T2DM did not add burden to their lives which contradicted with how the female participants felt. The females would take on the role as the breadwinners in the family if the men were incapable and alongside this, they would continue to play their domestic role in the house, which comforted the men.

The backbone of the family is my wife, while I worked in our shop. Now, not only do I suffer from diabetes, but I have also had a stroke. (I43; male, aged 58).

##### Lack of knowledge

Our study revealed that Indonesian T2DM outpatients lacked knowledge about how to manage their disease, mainly concerning complying with their treatment regimen. Some participants stated that they refrained from using chemical medicine because they had heard that the medicine (oral antidiabetic drugs) is toxic to the kidney. They believed that the continuous use of chemical medicine would lead to the increased risk of getting other diseases.

I am still motivated to take medicine because it is a lifelong treatment, but sometimes I do not want to because I am afraid of suffering from kidney disease. (I18; female, aged 60).

Some participants also reported that they followed advice from friends or colleagues, which had not been medically proven yet. They would only consume rice if it had been kept for a minimum of 24 h after being cooked with the assumption that recently cooked rice had a higher sugar content than rice which had been cooked the previous day.

My friend told me that T2DM patients could only eat yesterday’s rice because recently cooked rice has a higher sugar content. (I15, female, aged 73).

Another myth that the participants believed was to avoid sleeping between 7 and 11 in the morning. The participants said that they slept at night as usual, but waited until noon to take a nap if they felt sleepy in the morning. They believed that if T2DM patients slept in the morning, their blood sugar levels would increase.

To stay healthy, I first do some routine exercise in the morning, like taking a walk or cycling. Second, I do not sleep in the morning between 7 and 11 because people say that sleeping between those hours would increase my blood sugar levels. I only sleep afternoon. (I13, male, aged 60).

#### External diabetes distress

The DD related to external factors consisted of distress concerning healthcare services, diet, routine medication, monthly blood sugar checks, interpersonal distress (family) and financial concern. Indonesian T2DM participants preferred to receive medicine directly from their internist rather than from their GP. Before 1st January 2014, T2DM outpatients felt comfortable visiting their internists as part of their T2DM monitoring. Start from 1st January 2014, under the new national health insurance system, diabetes care was shifted to the primary care category (at least for initiating the care and medication). Participants complained that it was sometimes difficult to obtain a referral letter to the internist from their GP, even if it was just for a consultation.

##### Distress concerning healthcare services

Participants stated that they preferred to consult an internist about their disease rather than a GP because internists were viewed as having more profound and detailed knowledge compared to GPs. Few participants reported that the information they received from GPs duplicated what they had already learned from their internists. Internists not only stressed the importance of taking medication regularly and following a strict diet but also referred them to a nutritionist to consult on how best to adjust their meals to their condition. Several participants also stated that the internists’ psychological approach was much better.

During the consultation in the hospital, the internist asked, “Why does your blood sugar level increase?”I answered, “Maybe, it is because I think about it too much.” Then, the doctor said, “don’t think too much.” How can people live without thinking about certain matters? The internist then suggested that if I think too much, it could even have an impact on my heart and kidney. “Your husband and children will also be sad because they will think too much about your disease.” I got this advice from an internist. (I42; female, aged 37).

The majority of participants mentioned that consultation time was limited. Some participants forgot their doctor’s suggestions during the consultation after going home. The most common DD complaint was their confusion about the information provided by doctors.

The doctor did not provide a detailed explanation during the consultation. Therefore, after returning home, I was confused. (I32; female, aged 40).

Several participants had tried to conceal their real condition, especially in relation to uncontrolled blood sugar. Some participants said they tried to control their food consumption a day before going to a PHC or hospital so that the next day they would have lower blood sugar levels.

The doctor seemed angry at the level of my blood sugar when I visited. (I18; female, aged 60).

Many participants felt distressed about having to tell various doctors their T2DM story, including the diagnosis and all the drugs they had consumed. Varying opinions from different doctors were another source of distress, as indicated by the participants.

Every doctor has his/her opinion when I ask about sugar. One doctor told me that it is okay to drink a little sugar, while some strongly prohibit it. (I13; male, aged 60).

##### Diet

Complying with the strict diabetes diet to maintain the blood glucose is a challenging task for participants who are frequently involved in family gatherings or dinner parties. The most rational reason for this would be the difficulty in providing or asking the hosts to serve dishes suitable for the participants. On the other hand, some other participants seemed to be more at ease while on a diet because they argued that there would be nothing wrong as long as they consumed their medicines before the meal.

Sometimes, my family invites me to eat “sate gule kambing (mutton gulai)”. I realise this food can pose a threat to my health, but it is delicious. I think that it should not be a problem for me to eat sate because I took an insulin injection before eating. (I21; female, aged 65).

##### Routine medication

T2DM outpatients have to take a precise dose of medicine daily, and this was expressed as a source of distress. Further, this was mostly reported by participants who felt their families did not care about their emotional burden. They wanted their family’s support to remind them to take their medicine, at the very least.

At home, my children used to remind me to take medicine, but now my husband only reminds me if he is not out for a walk. So, when I am alone at home, I have to remember it myself. (I27, female, aged 72).

I live on my own, I have nobody, so I have to take care myself, including taking my medicine. Sometimes I forget to take medicine. Nobody reminds me. My child is far away. He is working in Jakarta. (I7, female, aged 67).

##### Monthly blood sugar checks

Almost all the participants said that the national health insurance programme, which provides them with a monthly blood sugar check, assisted them. Some of them nonetheless felt that a monthly check was insufficient. During the interview, three sources of distress related to the laboratory blood sugar checks were discovered. First, some informants were worried about missing their monthly blood sugar check appointment because they were busy (mostly male participants). Second, some female participants were worried about the results of their blood sugar check. They were worried that their blood sugar would be abnormal, especially in the group of participants who assumed that they had to take their medicine regularly for at least a month before. Another thing they worried about was the doctor being angry with them for having uncontrolled blood sugar.

If I do not get my blood sugar checked, I think about it all the time. (I33, male, aged 70).

Let’s put it this way. When a doctor sees that one month my blood sugar is low, but another it is high, the doctor usually gets angry. Once Dr. R was mad at me, but he is retired now. I let him get mad at me. I considered his being mad because of his loving and caring for his patients. (I1, female, aged 68).

##### Interpersonal distress (family)

Although the family support is one of the most significant factors in reducing DD, our study found that some female participants encountered some difficulties during the management of their T2DM treatment along with their dissatisfaction towards family member(s). Female participants, who are also housewives, felt the burden of preparing the food for the family as each family member has different tastes caused them distress. They felt trapped between their role as housewife and their dietary restrictions.

The family loves eating sweet dishes. They often love eating salty food. (I9; female, aged 57).

Another female participant stated her disappointment in her husband who did not seem to care to assist her during her T2DM therapy.

I was so heartbroken because my husband did not pay attention. Sometimes I asked him to get me insulin, but he refused, saying that he was busy working. Sometimes I almost lost my temper and ended up getting the insulin myself. (I40; female, aged 50).

The opposite situation occurred in married male T2DM participants as their wives acted as the prominent caregivers. The wives would provide all the husbands’ necessities to improve their T2DM treatment.

##### Financial concerns

The other complaint was related to the limited availability of drugs, either in terms of the quantity or the brands provided at the PHCs. The general assumption amongst participants seemed to be that branded drugs have better therapeutic effects compared to generic drugs, but they are more expensive. This was obviously not an issue for the economically stable, but it was a major problem for those with financial difficulties. Further, some participants with economic difficulties were frustrated that they had to follow the new regulations and consult with a GP first before being referred to an internist.

I use BPJS [the national health insurance], but I still cannot see the doctor whenever I like because I have to pay for it. (I42; female, aged 37).

I have to pay IDR 500,000 [USD 38] for a consultation with an internist [in private practice, outside BPJS], but I am so satisfied. (I38; female, aged 48).

#### Strategies for coping with internal diabetes distress

The strategies for coping with internal DD were spirituality, positive attitude, acceptance and getting more information about T2DM. The interview guidelines (Appendix 1) had no questions related to religion. However, most participants mentioned religion as a primary factor and the one with the most influence on their lives, especially when asked about the five questions related to emotional burden. It appeared from the interviews that acknowledging T2DM is a more common disease amongst older adults made coping with the disease and accepting being a T2DM outpatient easier.

##### Spirituality

Some participants described their T2DM as a warning from God for them to pay more attention to their health, especially to the types of food they consume. Older participants felt that T2DM brought them closer to God because they were encouraged and felt more inclined to go to a place of worship more often. The majority of the participants thought that communicating with God was the way to obtain comfort. Most participants in our study were elderly and understood that diabetes was common for their age. Some participants stated that one way to live with diabetes was to minimise stress.

Well, I never get angry when suffering from diabetes. I just feel grateful to God for having given me this diabetes. Diabetes is a gift from God to make me realise the need to manage my diet as well as to enjoy my life. (I1, female, aged 68).

##### Positive attitude

In addition to spirituality, another strategy adopted by the participants was to seek comfort in having a positive attitude. This meant that the participants tried to more actively remind themselves of the importance of keeping a positive mind set. For example, consciously believing that regularly taking their medicine and having monthly blood sugar checks in the PHC would result in better outcomes.

I am sometimes afraid that my blood sugar level will rise if I think about it. Then I tried to relax. I free my mind. I remind myself that I attend my monthly checks and take my medicine consistently. (I42; female, aged 37).

##### Acceptance

Another method of dealing with the disease was to accept living with T2DM. Some realised that thinking too much about T2DM would have negative consequences. The positive and negative effects of T2DM were subjective to each participant as it depended on their state of mind.

I do not think about how hard it is to live with T2DM. (I42; female, aged 37).

##### Getting more information about T2DM

One way the participants tried to improve their knowledge about T2DM was during their consultations with the doctor who managed them. Some participants felt their consultations were very short.

Every month, during every visit to the PHC, I always asked my doctor about the development of my diabetes. I would also recount my experiences after taking medicine. Even If I am just injured, I consult my doctor about it. (I1, female, aged 68).

Participants understood that exercise and a healthy diet were parts of their T2DM therapy. Most of the participants also reported the television as a source of information about T2DM. Another strategy was to join other T2DM programmes, such as T2DM club activities. In addition to being informed by doctors and internists, the clubs would also let them share their experiences with other T2DM patients. At these meeting, participants were also given the opportunity to ask questions about certain types of food and drinks that are recommended for T2DM; thus, clearing any discrepancy surrounding dietary allowance.

I know from my doctor that what we should avoid are […] sweet beverages and fatty meat. For example, when we go to ‘aqiqah’ (a Muslim social activity), goat meat is a type of food that should be avoided. (I33; male, 70).

I follow the advice for diabetes patients with the number of calories, schedule of eating and type of food. (I33, male, aged 70).

#### Strategies to cope with external DD

The coping strategies for external factors consisted of the following: healthcare support, traditional medicine, vigilance, self-management, social and family support and obtaining information about health insurance.

##### Healthcare support

Several current programmes were given to T2DM outpatients, such as weekly physical exercise and an awareness programme. These programmes are actively organised in some districts in Java by skilled practitioners and are supported by advanced facilities.

They have started “senam”(callisthenics) sessions with instructors. They give us a copy of the exercise routines. (I25; male, aged 67).

##### Traditional medicine

Traditional medicine was suggested as the best alternative to conventional medicine (or even the preferred ones) by most of the participants, especially by those disappointed with the effectiveness of oral antidiabetic drugs or insulin. For example, a female participant shared her experience of a time when she had needed a tooth removed. The dentist would not allow her to have it taken out because of her high glucose levels. This was stressful for her because she had both the pain from a toothache and the suboptimal effectiveness of conventional medicine. A colleague encouraged her to consume traditional medicine such as medicinal herbs, which immediately brought her blood sugar under control. Her tooth was extracted, and she felt very satisfied with the traditional medicine.

I still drink a herbal extract labelled “sarang semut” (Myrmecodia platyrea), but I do not tell the doctor (I3; female, aged 56).

In the morning I take glimepiride, after lunch I take metformin, and every night before bed, I drink five “sarang semut”. My blood sugar is now 99. I think therapy using “sarang semut” has halved my blood sugar. If my blood sugar is stable, I only drink one or two “sarang semut” to maintain it. (I3; female, aged 56).

Participants mentioned paying close attention to particular meals, for example, food with potential negative impacts on T2DM had to be avoided.

##### Vigilance

Unpleasant experiences also became strong moral lessons for some participants. These experiences were not only as a result of inappropriate dietary consumption, as several participants also learned from other people’s experience.

If my blood sugar level increases, I remember. Oh […] I should not drink sweet beverages. (I27; female, aged 72).

##### Self-management

Witnessing a dreadful incident related to T2DM, such as visiting a neighbour who had passed away because of T2DM or a foot amputation, was the most influential force for patients to pay closer attention to their T2DM treatment. Some participants reported that they were motivated to take their T2DM seriously after witnessing such incidents themselves, usually by trying to obtain as much information as possible to prevent T2DM complications.

My friend was harmed by diabetes. His thumb was amputated. Now he has passed away. It reminds me that I should be very careful about my diabetes. (I13; male, aged 60).

##### Social and family support

Spouses and children(s), as the closest family members, help participants directly. Showing affection, by reminding them to take medicine and helping them stick to their T2DM diet programme, helps optimise T2DM treatment management.

My husband warned me not to forget to take my medicine. (I42; female, aged 37).

When I eat with my husband, I remind him about the calorie content of each food we have. (I30, female, aged 55).

##### Obtaining information about health insurance

Some participants complained about the complicated health service bureaucracy. They worried about moving from one doctor to another. In the past, they had been very satisfied but were not accustomed to the new doctor.

I have to pay IDR 500,000 [USD 38] for a consultation with an internist [in private practice, outside BPJS], but I am so satisfied. (I38; female, aged 48).

## Discussion

For DD, our study identified two superordinate themes (diabetes distress and coping) and four subordinate themes (internal and external for each superordinate theme). Internal DD factors included disease burden, fatigue due to T2DM, fatigue not due to T2DM, emotional burden (fear, anxiety, etc.) and a lack of knowledge. Furthermore, external DD factors could take the form of distress concerning healthcare services, diet, routine medicine, monthly blood sugar checks, interpersonal distress (family) and financial concern. Internal coping strategies used to deal with DD included spirituality, positive attitude, acceptance and getting more information about T2DM. The external coping strategies were healthcare support, traditional medicine, vigilance, self-management, social and family support and obtaining information about health insurance. AS compared to the original DDS factors identified by Polonsky, et al. (emotional burden, physician distress, regimen distress and interpersonal distress) [24, 35], we identified various other factors such as distress concerning healthcare services and the tendency of Indonesian T2DM outpatients prefers to be handled by internists rather than by general practitioners.

Complaints on disease and emotional burdens were made by most participants, especially females. A study in Taif City, Saudi Arabia, of 509 participants (mean age 58 ± 14, 65% were males) reported that 54% of the total participants had moderate to high emotional distress with a higher percentage among female participants [36]. Besides gender differences, the study also revealed that higher levels of HbA1c, triglyceride, BMI, T2DM duration and interval between visits have a positive correlation with a higher score of DD [36]. Furthermore, another study of 119 participants (61% were Black), reported that emotional burden of diabetes was caused by the prescription of insulin [37]. Both of these studies do not only show DD, but they also agree that one of the recommended coping strategies was social support, especially family support [36, 37].

Fatigue due to T2DM emerged as a persistent symptom. This result of our research is in line with the review by Kalra and Sahay who stated that fatigue due to T2DM is closely related to DD, poor physical conditioning, less physical activities and less ability to self-manage diabetes [38]. A study done in the US of 48 T2DM participants (mean age 59.6 ± 7.2 years, 54% were males) revealed that a negative correlation between fatigue due to T2DM with HRQoL exists [38]. Moreover, in that research [38] it was stated that fatigue due to T2DM is related closely to quality of sleep, BMI and pain. Also, they found that male participants state that their fatigue was not caused by T2DM but by daily routine activities; as found in our dataset. So, there seems to be a different attitude towards fatigue between male and female participants.

In this research, we also found that the majority of participants reported distress concerning healthcare services. They were confused about the lack of socialisation of changes in the health insurance system. Since recently, all T2DM outpatient services must be started from primary care, including for those who have received in-tertiary care services. As an illustration, many elderly participants felt uncomfortable with changing from internists (in a tertiary care) to GPs (in a primary care). The results of this study can be a reference that besides requiring education about diabetes, T2DM patients in Indonesia are also recommended to obtain information about healthcare providers. A study in the US of 267 participants (mean age 58 ± 14, 56% were female) reported that lower scores of DD were significantly related to older age, lower BMI, healthy diet, higher self-efficacy, lack of knowledge and higher levels of healthcare provider support [10].

In our study, some participants began to better recognise their bodily condition, making them more aware of T2DM therapy and diet. When they felt that their blood sugar was rising, some of them would identify the type of food they had just eaten or remember their medication schedule. Continuous vigilance in monitoring should not only be the initiative of patients, but doctors and family of patients also need to play a role in paying attention to this condition so that the goals of therapy can be achieved. As an illustration, when patients report certain conditions during their therapy, for example feeling dizzy, nauseous or other symptoms, doctors can give considerations to review medication regimens and analyse utilisation of appropriate medications [39] or by changing the medicines. Related to family support, some participants stated that their spouses or children greatly helped to improve T2DM self-management practices. This condition is also described in a qualitative research study in 20 T2DM participants in Peru, where spouses or children encourage and motivate them to provide instrumental support by empowering them to fight for their health, improve healthy meals and share physical activity [40]. All in all, our study and those cited illustrate the core issue of an optimal interplay between patients, family, doctors, pharmacists and health systems to confront diabetes and DD.

One of the main findings concerned spirituality as a coping strategy. The term spirituality has been formulated as Puchalski et al. “Spirituality is the aspect of humanity that refers to the way individuals seek and express meaning and purpose and the way they experience their connectedness to the moment, to self, to others, to nature, and to the significant or sacred” [41]. It has been noted before that there is a strong positive correlation between spirituality and coping with chronic disease [42]. Furthermore, spirituality is also linked to distress, confusion, depression, quality of life [43] and the providence of motivation and positive attitude change [44]. Mostly, our participants believed that the disease was their destiny and that they should accept it. A female participant even stated,” diabetes is a gift from God.” It illustrates how spirituality may be related to religion in this specific Indonesian setting as the country with the world’s largest Muslim community. A qualitative study on 45 women with T2DM also concluded that spirituality was significantly related to coping with and cognitively reframing DD [45]. It also had positive effects on their blood sugar levels [46]. As a coping mechanism, the women adapted their daily activities to reduce the burden of T2DM. These findings are similar to the results of studies conducted amongst elderly Malaysian Muslims [27, 47]. Another study found a positive correlation between spiritual service attendance (contact with spiritual leaders) and controlled blood sugar levels in T2DM patients [48]. Finally, our study highlights the importance of accepting one’s self to help cope with DD. A study in San Francisco found that education and providing an understanding of self-acceptance and commitment will positively impact self-management behaviour and achieve controlled HbA1c targets [49].

In our study, female participants reported having a higher level of DD compared to male participants. This result was similar to research on 815 T2DM patients in Eastern Massachusetts treated in primary care [50]. Previous research has suggested differences in attitudes between males and females in responding to diabetes, with females potentially being more sensitive to their illness [51, 52]. Another study stated that there were five factors of explanation for gender differences in health [53], which were: (i) biological risks of disease, (ii) acquired risks of illness and injury, (iii) psychosocial aspects of symptoms and care, (iv) health reporting behaviour and (v) prior health care. Male participants might be more unaware of the symptoms and optimise socialisation with the people around them in order to ignore their physical discomforts [51]. However, our analysis differed from the study of 51 Australians with T2DM treated in primary care, in which the level of DD in female participants in this study was not significantly different from male participants [54]. Ergo, further research is needed to confirm these issues.

Another main finding in our research was the importance of feeling and stating the responsibilities as a housewife. Being a housewife entails food preparation not only for themselves but also for their families who might have very different tastes and may not want to follow a diabetes diet. This may cause specific tension within the family. A study of 185 Iranians with T2DM (85 are housewives) found that nearly 50% of the total number of housewives reported that they experienced DD [14]. It further showed that Iranian housewives who spent most of their time at home, besides fulfilling their other responsibilities, also worried about the possibilities of T2DM complications occurring [14]. Another study stated that there is currently a change in hierarchal structure in the family in which women/housewives are taking a central role especially in taking responsibility for family health [55]. In summary, “being a housewife” means that besides from attending daily responsibilities, they are also responsible for the health of the other family members and diabetes may significantly complicate this.

The overall findings of this study show that Indonesian T2DM outpatients need to attend to their psychological needs in addition to their physical needs (adequate medicine, laboratory and consultation time) in order to optimise their T2DM treatment. How doctors communicate in providing advice positively affects their emotional state. Research into T2DM patients in primary care settings in eighteen countries revealed that limiting patient consultation time increases the risk of DD 35-fold [56]. Furthermore, distress concerning the healthcare service was the most commonly reported factor in the FGDs. Within this category, participants not only focused on physician distress but also emphasised issues with the health insurance service bureaucracy. Some remarks on traditional medicine may trigger the desire to further investigate the role of traditional medicine and the need for education in this area as well.

Some limitations in this study have to be acknowledged concerning the fact that data were only collected from one PHC in Surabaya, which has better facilities and better health personnel in comparison to several other PHCs in remote areas. Furthermore, it is important to note that only two participants participated in the depth-interviews process, limiting generalisability of those findings. In this study, we also did not look into the relationship between T2DM duration and DD. This was because throughout the data collection process the majority of the participants stated that they did not know when they first suffered from T2DM. As an illustration, several participants admitted that they just realised they had T2DM when they went to the dentist and a tooth extraction process had to be aborted because of the high blood sugar. Another participant admitted that in her case, it was not until she found out that she had breast cancer and required surgery, but the operation had to be postponed due to the high blood sugar. Another group of participants also stated that they did not have a routine blood sugar test because they assumed that they had no one in the family with T2DM. It has also been reported previously that the majority of Indonesian patients visits health facilities only after their diabetes has gotten worse or is accompanied by T2DM complications [57]. This stresses the relevance of T2DM screening to become one of the Indonesian government’s priorities, especially in its drive to strengthen primary healthcare services throughout Indonesia.

Our study’s strength lies in the detailed reasoning obtained in all four DD domains compared to other DDS studies. Our study showed a high participation rate of 86% in terms of the FGDs and in-depth interviews. Also, our findings are more detailed than the original DDS studies which did not specify the type of physician distress [24, 35]. As an illustration, we could detect that T2DM outpatients felt better assured if they were treated by internists rather than by GPs. Finally, with our level of detail, we could assess that spirituality is an important coping mechanism to reduce diabetes distress and that specific challenges exist for housewives with T2DM.

## Conclusion

Our study shows that for Indonesian T2DM-patients, spirituality, having a positive attitude and acceptance are the most common coping mechanisms for reducing DD. Furthermore, our study revealed an overall positive attitude towards dealing with T2DM as well as a need for more information about T2DM and potential coping strategies. Finally, an important finding of ours relates to differences in DD between males and females, and potential DD associated with health services provision and the specific challenges faced by housewives with T2DM.

## Appendix: data collections guidelines

We questioned to all the participants based on 17 questions listed in the DDS questionnaire, [35] as follows:

1. Feeling that my doctor does not know enough about diabetes and diabetes care. Does it become a problem for you? For example, if the doctor does not provide you with a clear explanation, does that cause problem? What is your experience with consultations with the doctor, so far?
2. Feeling that diabetes is taking up too much of my mental and physical energy every day. For example, you feel stressed because of thinking about living with diabetes. Have you ever been so tired? Have you ever felt such feelings?
3. Not feeling confident in my day-to-day ability to manage diabetes. For example, to maintain your dietary and sanitary habits, to take medicine on time and to do regular physical exercise. Do you feel confident that you can do all the activities involved in diabetes treatment?
4. Feeling angry, scared and depressed when I think about living with diabetes. Do you feel that way?
5. Feeling that my doctor does not give me clear enough instructions on how to manage my diabetes. It is because the doctor does not provide clear explanations during the consultation so that after returning home, you get confused about what dosage of medicine you have to take: how many pills you have to take in a day, for instance? Should they be taken before or after a meal? Have you experienced this? In your opinion, is this a problem for you?
6. Feeling that I am not testing my blood sugar frequently enough. So, for example, you have previously frequently undergone blood sugar checks. If you find yourself unable to do this for some reason, do you feel worried/anxious?
7. Feeling that I will end up with serious long-term complications, no matter what I do. Complications due to diabetes which are incurable include stroke, heart failure or renal failure. Do you keep thinking about these complications despite having followed your diet and taken medicine regularly?
8. Feeling that I am often failing in my diabetes routine. For example, you have to do physical exercise a minimum of twice a week, follow your strict diet and not forget to take your medicine. Have you ever failed to do these activities? Another common example is a failure in your diet. You overeat at a wedding reception. Have you ever experienced this?
9. Feeling that friends or family are not supportive enough of self-care efforts (e.g. planning activities which conflict with my schedule or encourage me to eat the ‘wrong’ foods).For example, when eating out with your spouse, who does have diabetes, and the dishes ordered do not fit your diet. Is that a problem for you?
10. Feeling that diabetes controls my life. You can only consume limited types of foods due to diabetes. Does this limitation cause you any problems?
11. Feeling that my doctor does not take my concerns seriously enough. When you tell your doctor about your anxiety at the possibility of having complications, the doctor seems to pay less attention to you. Do you feel that the doctor does not respond to your complaints seriously? Is that a problem for you?
12. Feeling that I am not sticking closely enough to a healthy diet. For example, for women who have to prepare delicious meals for their families. The children ask for curry with thick coconut milk. Is this a serious problem for you?
13. Feeling that my friends or family do not appreciate how difficult living with diabetes can be. For example, your friends and family want to go out for a meal with you. You know they are going to order some delicious dishes which you cannot eat. It thus seems that your friends and family ignore your condition. Have you ever felt this way?
14. Feeling overwhelmed by the demands of living with diabetes. You have to take medicine every day, do regular physical exercise and get regular check-ups from your doctor. You also have to control your nutrient intake and eating times. Do these activities overwhelm you?
15. Feeling that I do not have a doctor who I can see regularly enough about my diabetes. Thisis different from the regular monthly consultation you usually have. It means that you want a longer consultation with the doctor about various complaints such as paraesthesia, hypertension or other medical conditions related to T2DM.
16. Not feeling motivated to maintain my diabetes self-management. For example, you depend on medicine, diet and regular physical exercise, and you feel less motivated to continue these daily routines because T2DM treatment is for life. Another example that you might find easier to relate to is that you cannot be bothered to take your medicine and do physical exercise. Have you ever felt this way?
17. Feeling that friends or family do not give me the emotional support I would like. For example, they seldom remind you to stick to your T2DM treatment regime.
